# Carbon Allotropes-Based Paints and Their Composite Coatings for Electromagnetic Shielding Applications

**DOI:** 10.3390/nano12111839

**Published:** 2022-05-27

**Authors:** Ioan Valentin Tudose, Kyriakos Mouratis, Octavian Narcis Ionescu, Cosmin Romanitan, Cristina Pachiu, Emil Pricop, Volodymyr H. Khomenko, Oksana Butenko, Oksana Chernysh, Viacheslav Z. Barsukov, Mirela Petruta Suchea, Emmanouel Koudoumas

**Affiliations:** 1 Center of Materials Technology and Photonics, Hellenic Mediterranean University, 71410 Heraklion, Crete, Greece; tudose_valentin@yahoo.com (I.V.T.); kmuratis@hmu.gr (K.M.); 2 Chemistry Department, University of Crete, 70013 Heraklion, Crete, Greece; 3 National Institute for Research and Development in Microtechnologies (IMT-Bucharest), 023573 Bucharest, Romania; onionescu@gmail.com (O.N.I.); cosmin.romanitan@imt.ro (C.R.); cristina.pachiu@imt.ro (C.P.); 4 Petroleum and Gas University of Ploiesti, 100680 Ploiesti, Romania; emil.pricop@upg-ploiesti.ro; 5 Department of Electrochemical Power Engineering and Chemistry, Kyiv National University of Technologies and Design, 01011 Kyiv, Ukraine; v.khomenko@i.ua (V.H.K.); keeh@knutd.edu.ua (O.B.); chernyshoksana14@gmail.com (O.C.); 6 Department of Electrical and Computer Engineering, Hellenic Mediterranean University, 71410 Heraklion, Crete, Greece

**Keywords:** EMI shielding applications, alcohol-based conductive paints, multicomponent nanocomposites, carbon-based materials

## Abstract

The present manuscript reports on optimized formulations of alcohol-based conductive paints for electromagnetic interference shielding (EMI), which can ensure compatibility and reduce the visibility of electronic equipment, as a continuation of our previous work in this field, which examined water-based formulations for other applications. Graphite, carbon black, graphene, Fe_3_O_4_, Fe ore, and PEDOT:PSS in various ratios and combinations were employed in an alcohol base for developing homogeneous paint-like fluid mixtures that could be easily applied to surfaces with a paintbrush, leading to homogeneous, uniform, opaque layers, drying fast in the air at room temperature; these layers had a reasonably good electrical conductivity and, subsequently, an efficient EMI-shielding performance. Uniform, homogeneous and conductive layers with a thickness of over 1 mm without exfoliations and cracking were prepared with the developed paints, offering an attenuation of up to 50 dB of incoming GHz electromagnetic radiation. The structural and morphological characteristics of the paints, which were studied in detail, indicated that these are not simple physical mixtures of the ingredients but new composite materials. Finally, mechano-climatic and environmental tests on the coatings demonstrated their quality, since temperature, humidity and vibration stressors did not affect them; this result proves that these coatings are suitable for commercial products.

## 1. Introduction

The revolution of the new 5G technology is ongoing, and it comes with some significant challenges regarding an increasing level of electromagnetic background noise as a result of wireless connections between intelligent sensors, actuators, and numerous associated routers. These conditions increase electromagnetic interference, and special measures will be required to prevent the problems coming from this phenomenon. As is known, electromagnetic interference (EMI) can disrupt electronic devices, equipment, and systems used in critical applications. Examples, to name a few, include medical, military, and aerospace electronics, mass transit systems, industrial touch screens, and navigation and vehicular control systems. The causes of electromagnetic interference are numerous and based on both manufactured and natural radiation sources. The results can range from temporary disturbances and data losses to system failure and even loss of life, since EMI also affect humans, animals, and the environment. Therefore, EMI shielding materials are required, materials such as flexible metal screens, metal wires, and metal foams. Coatings made of metallic inks can be also applied to the interiors of electronic enclosures to provide an EMI-shielding solution. Each of these shielding methods has its advantages, but lightweight paint-like carbon-based coatings can combine the electrical properties of metal with excellent mechanical material properties at a lower cost and easier application. In very recent and extensive reviews on this subject [1,2,3,4,5,6,7,8,9,10,11,12], many nanomaterial-based potential solutions have been developed and tested for specific electromagnetic-shielding applications. Among these, different forms of carbon, such as graphene, carbon nanotubes, carbon fiber, carbon aerogels, carbon black, activated carbon, and carbon nanoparticles, as well as their hybrid composites, have been widely advanced and considered for EMI shielding in the GHz range [4,5,10,11,12,13,14,15,16,17]. The electrical and dielectric properties, as well as the shielding effectiveness of various polymer/graphene nanoplatelets (GNP) polymer/carbon black (CB) nanocomposites, were investigated recently [5,7,8,9,10,11,12,13], but multiple component formulations that can result in hybrid composites based on carbon-allotrope conductive fillers are not so frequent. Hybrid composites based on carbon-allotrope conductive fillers are very important for the current developments due to the new 5G technology that requires improved EM-shielding properties, particularly in the highest bands of frequencies needed for wireless applications. The combination of the microstructures of these carbon materials as building blocks of the shielding layers, as well as the inclusion of magnetic and dielectric materials in the shield, proved to be helpful for EMI-shielding effectiveness [2,13].

EMI shielding regards the attenuation of an incident EM radiation by reflection and absorption by a material that would act as a barrier against the penetration of the radiation into a system. The reflection loss links to the interaction between the incident wave and mobile electric-charge carriers and the impedance discrepancy at the interface of the shielding material. The absorption loss is associated with the dissipation of electromagnetic-wave energy into the shielding materials due to heat loss under the interaction of the electric dipoles in the material and the incident EM radiation. The present paper reports the most recent achievements in a larger research trial that is dedicated to the development of efficient and environmentally friendlier multicomponent paints for EMI-shielding purposes. Previously, we reported the successful fabrication of water-based formulations of EMI-shielding effective paints [13] for use on hydrophilic surfaces and the compositional optimization of the CB-based solid-state composites formulations for further integration in the EMI-shielding paints [18].

This study focuses on fabricating homogeneous alcohol-based paint-like fluid mixtures, easily applied onto surfaces with a paintbrush; the application of these mixtures leads to homogeneous, uniform, opaque layers that dry fast in the air at room temperature and have quite good electrical conductivity, which can offer efficient EMI-shielding performance on polymeric and metallic surfaces. Various ratios of carbon-based materials and different preparation parameters were tested so that effective shielding paints can be obtained by taking into account an optimum combination of physical/chemical properties and shielding performance. As a result, hybrid composites based on carbon-allotrope conductive filler paints with optimized properties were developed; these paints both offer uniform, homogeneous, and conductive layers with a thickness up to 0.5 mm without deformation and cracking and exhibit a shielding effectiveness of up to −50 dBs for electromagnetic radiation in the GHz frequency range. The structural and morphological characteristics of these paints were studied in detail. These simple paint-like coatings can become an excellent choice for product designers who need to meet various sealing and insulation challenges.

## 2. Materials and Methods

The electromagnetic-shielding layers were deposited on thin paper placed directly on a metallic frame, designed especially for the waveguide used, by brushing paint-like dispersions. Graphene nanoplatelets (GNPs), natural graphite, carbon black, magnetite (Fe_3_O_4_), iron ore (Fe ore), poly (3,4-ethylenedioxythiophene) poly (styrene sulfonic acid) (PEDOT:PSS) and polyvinyl butyral were mixed into an alcohol base for developing the paints as follows. For the preparation of the B1, B2, B3 paints, battery-grade graphite GAK-1 from Zavalie Graphite Co. (Kiev, Ukraine) and graphitized carbon black (PUREBLACK^®^) (PB) (Superior Graphite Co., Chicago, IL, USA) were used. Natural flake graphite GAK-1 relates to coarse-grained graphite with an average particle size of 132 microns and provides the paint with electrical conductivity on a sufficiently high level. To prepare V paints, large grains of pure natural graphite (EMFUTUR Technologies Ltd. Spain) were milled using a ball mill and dimensionally separated using a 0.02 microns sieve. Graphene nanoplatelets (EMFUTUR Technologies Ltd., Villarreal, Spain) (GNPs), carbon black (CB), poly (3,4-ethylenedioxythiophene) poly (styrene sulfonic acid) (PEDOT:PSS) purchased from Heraeus Germany and polyvinyl butyral PVB (Sigma, Roedermark, Germany) were also used. The compositions of the paints, based on preliminary compositional studies reported in [18] and on an initial parametric investigation, were as following:

⮚ B1: 60% GAK-1, 20% PB and 20% PVB
⮚ B2: 50% GAK-1, 16.7% PB, 16.7% Fe_3_O_4_, 16.7% PVB
⮚ B3: 50% GAK-1, 16.7% PB, 16.7% Iron Ore, 16.7% PVB
⮚ V: 25% natural graphite, 25%GNPs, 25% Fe_3_O_4_, 12.5% CB, 7.5% PEDOT:PSS, 5% PVB

The ingredients were carefully weighed and mechanically mixed to obtain a homogeneous suspension with adequate rheological properties to be used as paint. Various preliminary trials were performed to achieve the strength requirements in practical applications, such as peel strength and tensile strength. The formulation was successively adjusted until the fluid mixtures became suitable as paint. The uniformity of the paint thickness was achieved by using a controlled quantity of dry substance in a specific volume. Given the paint homogeneity, the thickness would be approximately the same by applying the same amount of paint on a particular surface.

### Characterization Methods

The obtained materials were characterized by SEM, XRD, and Raman Spectroscopy, and their electrical and shielding properties were evaluated.

Scanning electron microscopy (SEM) characterization was performed with a W filament LV6064 SEM (Jeol company, Tokyo, Japan) in a high vacuum, in order to investigate and understand the formation and the architecture of the obtained nanocomposite materials. All samples were characterized in the high-vacuum mode without any conductive coating. X-ray-diffraction (XRD) investigations were performed using a Rigaku Ultra high-resolution triple-axis multiple reflection SmartLab X-ray Diffraction System (Osaka, Japan) in grazing incidence geometry, varying the 2θ from 5 to 50° with a speed of 4°/min. The peak indexing was achieved using ICDD (International Center for Diffraction Data) database. Raman analysis was done using a Witec alpha 300S Gmbh Germany system, with an Nd-YAG laser at 532 nm and confocal Raman microscopy (high-resolution confocal Raman imaging, AFM and SNOM). Finally, the electrical resistance of the nanocomposite samples was determined with a FLUKE 8846A (Fluke Electronics, Everett, WA, USA) multi-meter using the four-point configuration [17].

The shielding performance of the developed materials was examined and presented in our previous study [13], in terms of shielding effectiveness, a parameter that depends on several factors related to both the material and the design used; this parameter can be expressed as:

$$SE=10\log\left(\frac{P_{i}}{P_{t}}\right)=20\log\left|\frac{E_{i}}{E_{t}}\right|$$

where *P_i_* is the incident and *P_t_* the transmitted wave, *E_i_* and *E_t_* are incident and transmitted electric fields, respectively.

The absorbance (A_b_) of the radiation could be calculated by measuring the reflectance (*R_e_*) and the transmittance of the material; this measurement can be obtained with the following formula:

A_b_ = 1 − *T_r_* − *R_e_*

where $R_{e}$ is the reflectance and $T_{r}$ is the transmittance of the material

$$R_{e}={\left|\frac{E_{r}}{E_{i}}\right|}^{2}={\left|S_{11}orS_{22}\;\right|}^{2}$$

$$T_{r}={\left|\frac{E_{t}}{E_{i}}\right|}^{2}={\left|S_{12}orS_{21}\;\right|}^{2}$$

are the scattered parameters [18] and could be measured with a Vector Network Analyzer (VNA).

**Figure 1:**
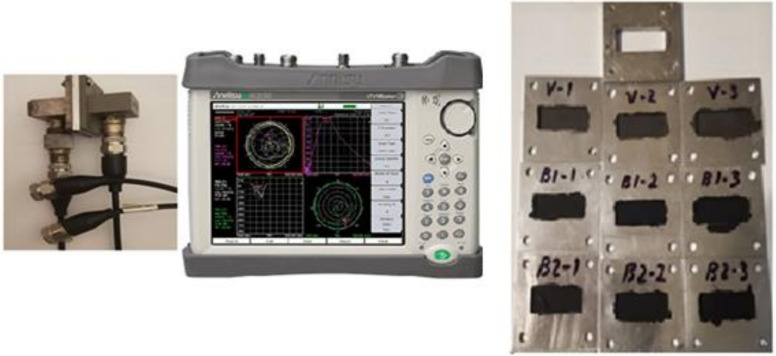
The measuring setup for EMI-shielding efficiency based on the ANRITSU Vector Network Analyzer.

The measuring setup used for the determination of the shielding performance of the developed paints is presented in Figure 1, and it was based on the portable vector network analyzer Anritsu MS214C: 9 kHz–6 GHz (Anritsu Co., Ltd., Tohoku, Japan), two Waveguide to Coax Adapters, and a diaphragm (holder for sample). The waveguides had a cut-off frequency of 4.3 GHz, resulting in a measurement range between 4.3 and 6 GHz. With this setting, the accuracy of measurements was the highest possible, since they were not affected by any interferences.

The reference measurement was conducted with an empty holder at the beginning of the experiments. In addition, five markers (4.398 GHz, 4.588 GHz, 5.079 GHz, 5.541 GHz, 5.900 GHz) were set in order to perform better measurements in points of interest.

**Figure 2:**
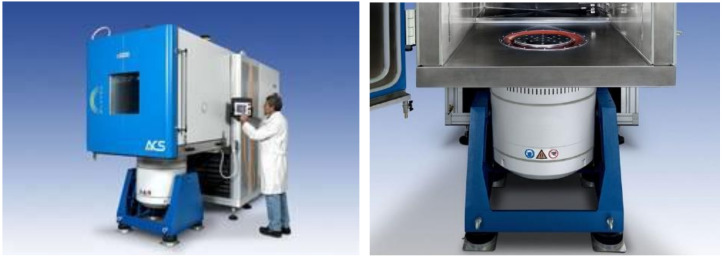
Angelantoni CH250 climatic chamber + TIRA S55240/LS—Vibration system.

The tests for potential commercial applications were conducted for two of the developed paints, which presented the best absorption properties. The behaviour of the painted surface was environmental and mechano-climatic tested in an Angelantoni CH250 climatic chamber + TIRA S55240/LS—Vibration system, presented in Figure 2. The tests were performed according to the referenced documents [19,20,21,22,23]. 

## 3. Results and Discussion

### 3.1. SEM Characterization

**Figure 3:**
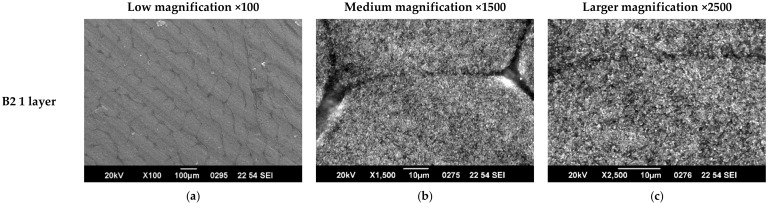

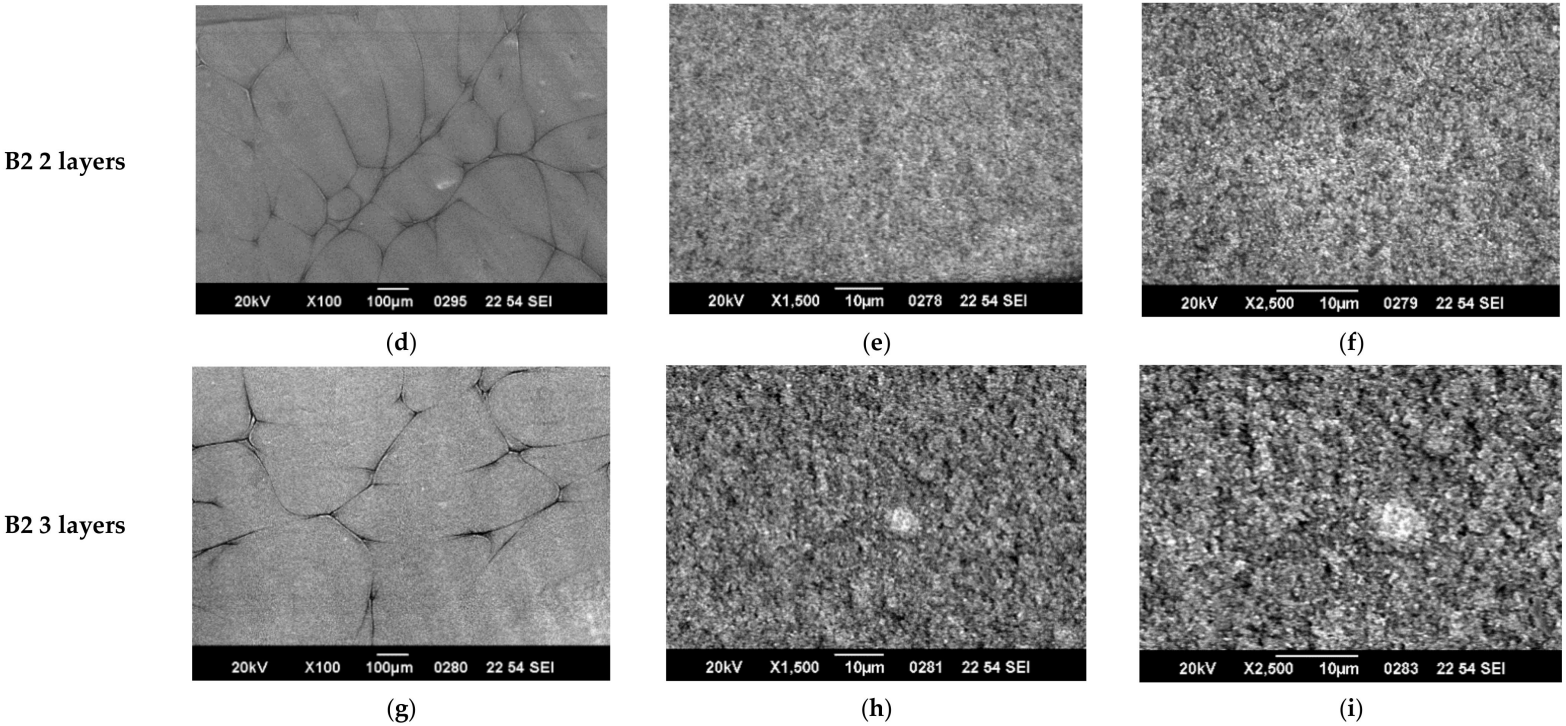
SEM characterization of 1, 2 and 3 layers of paint B2 at different magnifications. (**a**) B2.1 ×100 (**b**) B2.1 ×1500 (**c**) B2.1 ×2500 (**d**) B2.2 ×100 (**e**) B2.2 ×1500 (**f**) B2.2 ×2500 (**g**) B2.3 ×100 (**h**) B2.3 ×1500 (**i**) B2.3 ×2500.

**Figure 4:**
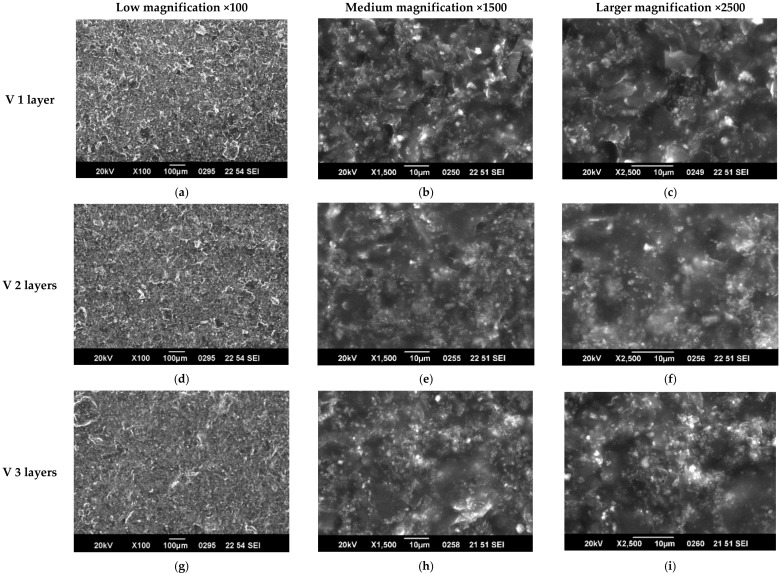
SEM characterization of 1, 2 and 3 layers of paint V at different magnifications. (**a**) V1 ×100 (**b**) V1 ×1500 (**c**)V1 ×2500 (**d**) V2 ×100 (**e**) V2 ×1500 (**f**) V2 ×2500 (**g**) V3 ×100 (**h**) V3 ×1500 (**i**) V3 ×2500.

Examples of SEM characterization of 1, 2, and 3 layers of paints, respectively, in either B2 or V are presented in Figure 3 and Figure 4. As one can observe from the low magnification (×100) images in Figure 3, paint B2 forms discontinuous layers characterized by some domains. This is a common characteristic of all 3 B formulations. The domains boundaries may be preventing the electrons’ flow, leading to lower conductivity of the layers; therefore, lower conductivity is expected. The medium and larger magnification images show that quite homogeneous layers are formed that become more compact and uniform as the number of layers increases. In the case of V formulation, it can be observed that the coatings are homogeneous and uniform in all the cases and that surface morphology is less affected by the number of layers. The crisp contrast of images (characteristic of surfaces formed of regions with very different electric conductivity, the insulating regions prevent electrostatic charge discharge to ground) in the B2 paint formulation shows that the composite components’ conductivity is quite different, and charge accumulation is promoted on the surface. In the case of the V formulation, the coating shows a more uniform conductivity of components in the composite.

**Figure 5:**
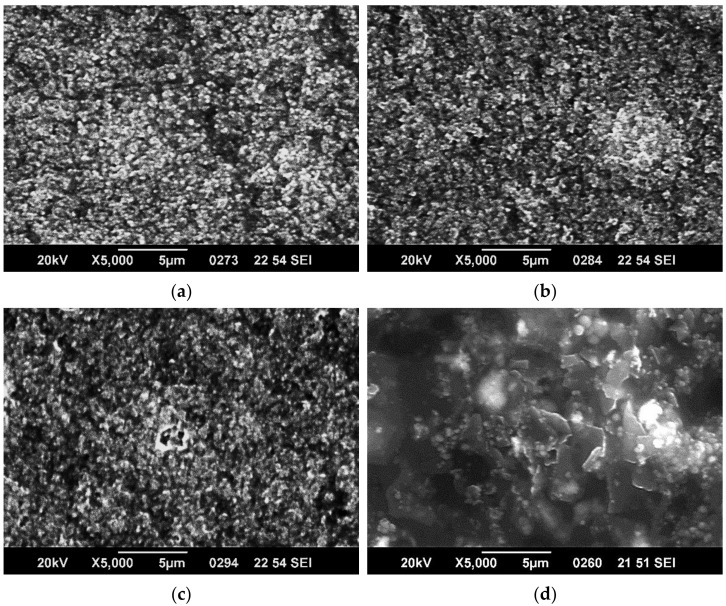
×5000 magnification SEM images of 3 layers of paint (**a**) 3 layers of B1 paint (**b**) 3 layers of B2 paint (**c**) 3 layers of B3 paint (**d**) 3 layers of V paint.

The above observations are more evident from the SEM images at magnification ×5000 presented for the 3 layers of all coatings in Figure 5.

SEM characterization can explain the similar electric and EMI-shielding properties of B2 and B3 samples that were observed as well as the enhanced performances of V samples, in which GNPs seem to act as electrical connectors of the other paint components. The effects of the discontinuities characteristic of all “B” composites on EMI-shielding properties cannot be assessed, and further studies regarding the multicomponent composites structuring are ongoing.

### 3.2. XRD Characterization

**Figure 6:**
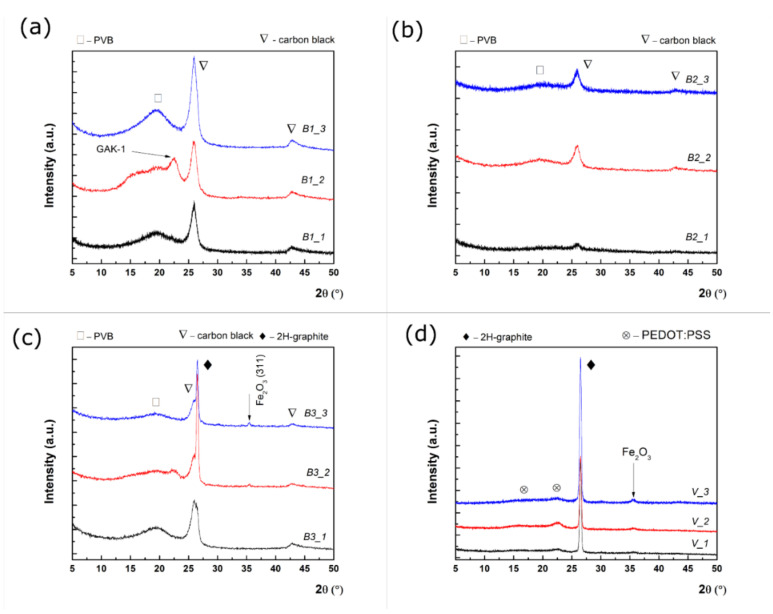
GIXRD patterns for (**a**) B1, (**b**) B2, (**c**) B3, (**d**) V sample.

Grazing incidence X-ray diffraction was employed to study the microstructure of the obtained nanocomposites for one (_1), two (_2), or three layers (_3) of all the paints studied. The peak identification was made with International Crystallography for Diffraction Data (ICDD) database. GI-XRD patterns for B1, B2, B3, and V samples are shown in Figure 6a–d.

It is known that black carbon exhibits two diffraction peaks at 25.9° and 42.8°, which correspond to an interplanar distance of 0.35 nm and 0.21 nm, respectively [24], which can be seen in Figure 6a–c. In addition, a broad diffraction feature was observed below 20°, indicating that PVB was a partially crystalline polymer [25]. The presence of a diffraction feature at 22.7° for two layers of paints should be noted, which would be ascribed to the battery-grade graphite (GAK-1) with an enlarged interplanar distance (e.g., 0.39 nm) than that of the pure graphite (e.g., 0.33 nm) [26]. Similarly, B2 samples present diffraction peaks characteristic of PVB and black carbon without any trace of iron oxide, since this was used at a low concentration. Further, the XRD pattern for B3 indicates the coexistence of graphite and black carbon. One can observe that the (0002) hexagonal graphite reflection (ICDD card no 041-1487) possesses a superior crystallinity to the black carbon. According to the relative intensity ratio (RIR) analysis, the relative intensity of graphite against black carbon increases from 47% (one layer) to 68% (three layers) as the number of layers of paint increases. At the same time, a small diffraction feature occurred at two and three layers, ascribed to Fe_2_O_3_ (hematite phase ICDD card no. 33-0664) at 35.5°, and this feature can be observed. Since a magnetite phase was used as precursor in the composite preparation, one can assume that the conversion of Fe_3_O_4_ to Fe_2_O_3_ occurred while mixing with the other components. The small Full Width at Half Maximum (FWHM) of the (0002) reflection of the graphite proves the high degree of crystallinity, reflected in the large value of the mean crystallite size. According to the Scherrer’s equation, the mean crystallite size for graphite is 24 nm, 21.5 nm, 23.5 nm at 1, 2, and 3 layers of paint. This equation relates the mean crystallite size (τ) to the FWHM, β of the diffraction peak, in the following way [27]: τ = kλβcosθ, where k is a shape factor taken as 0.9, while θ is the angular position of the evaluated diffraction peak. A significant broadening can be observed for black carbon for the B1 and B2 samples (FWHM~1.24°), indicating that the black carbon crystalline domains are around 7 nm. Finally, the V samples present a narrow diffraction peak attributed to graphite at 26.44°, two broader diffraction features at 16.4° and 22.4° assigned as PEDOT:PSS (card no. 47-1748), as well as a small diffraction feature given by Fe_2_O_3_. XRD characterisation suggests that the increase of thickness improves the coating quality for all four paints, and it proves that the coatings are not simple physical mixtures of the ingredients but new composite materials. 

### 3.3. Raman Spectroscopy Characterisation

**Figure 7:**
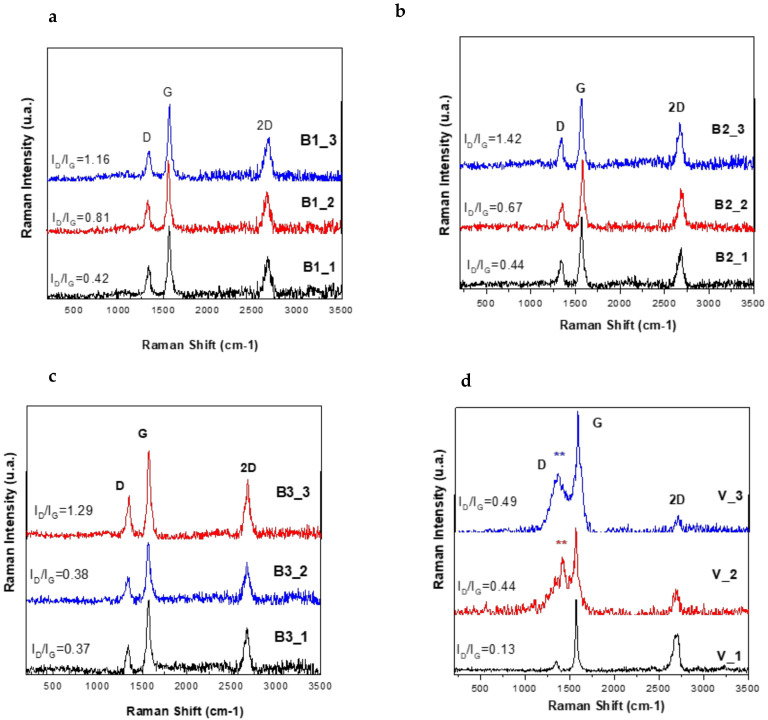
Raman spectra of 1, 2 and 3 layers of (**a**) B1 (**b**) B2, (**c**) B3 and (**d**) V paints.

Figure 7 shows Raman spectra of 1, 2 and 3 layers of the (a) B1 (b) B2, (c) B3 and (d) V paints. The Raman spectra of all samples show three distinct Raman peaks for the D band (disordered graphitic site sp3) at around 1384 cm ^−1^, G band (graphitic site sp2) at around 1518 cm^−1^, and 2D band at 2680 cm^−1^, which indicate the number of stacked graphitic layers respectively [28].

The other way to recognize the signature structural characteristics of the stacked layers is the ratio IG/I2D, where the peak intensity is denoted as I(D), I(G), and I(2D) for the D, G, and 2D peaks, respectively (Figure 7). For measuring the ID/IG in the carbonic materials, in the literature two methods are used: measuring the area under the curve by peak fitting (Integration of peaks function in Origin Program) for broader peaks, or the direct intensity is used to calculate the ratio when the peaks are sharp. The ratio IG/I2D in this case was estimated by extracting the intensity values with two decimals for de D-band and G-bands in Origin 8.5 program from the processing of numerical data of the Raman-spectra acquisition in Witec software. In the case of samples B1-B3, the IG/I2D ratio is higher than 1, which may be due to the stacking-like multilayer arrangement of graphitic structure in the PVB polymer matrix. [26]. The ratio of the intensities (ID/IG) that provide the information on graphitic material’s structure and domain size was calculated for all the samples. ID/IG increases with an increasing defect density for low and moderate defect density. ID/IG is markedly increased in contrast with the graphitic sample B1, suggesting the formation of some sp3 carbon by functionalization with Fe_3_O_4_ in the B2 sample and Iron Ore in the B3 sample [29,30,31]. These values give only a qualitative insight into the structure of multicomponent composite materials. For an accurate study, further experiments are ongoing.

The characteristic peaks of C-H stretching vibration and CH_2_ bond of the PVB and PEDOT:PSS hydrocarbon backbone are clearly visible at 2942 cm^−1^ (*) and 1421 cm^−1^ (**), respectively, in the V samples [13].

Raman spectroscopy results confirms once more the fact that the hybrid composite coatings are not a physical mixture of components but new materials with particular properties. 

### 3.4. Resistance Measurements

The measured values of the electrical resistance are presented in Table 1. As one can observe, the samples with 3 layers of paint show the lowest electrical resistance for all the formulations. This can be attributed to the better structuring of the composite material as observed from the composite layers’ SEM observed characteristics.

### 3.5. Shielding Properties

**Figure 8:**
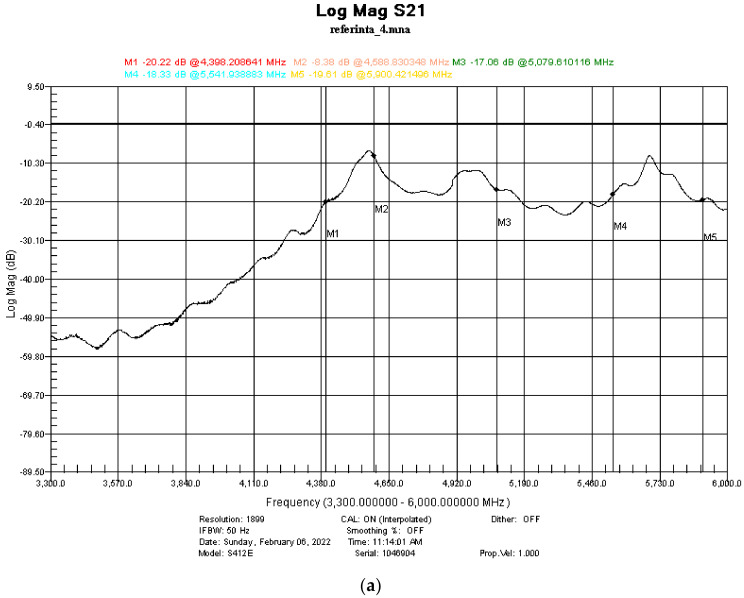

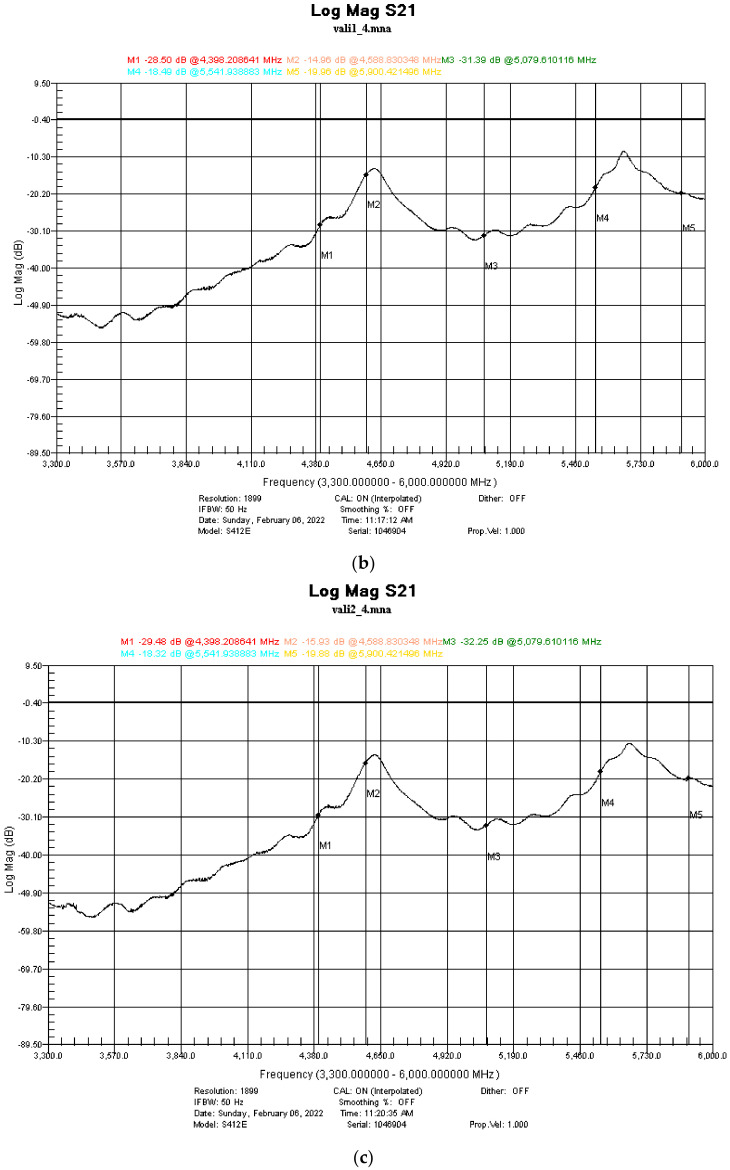

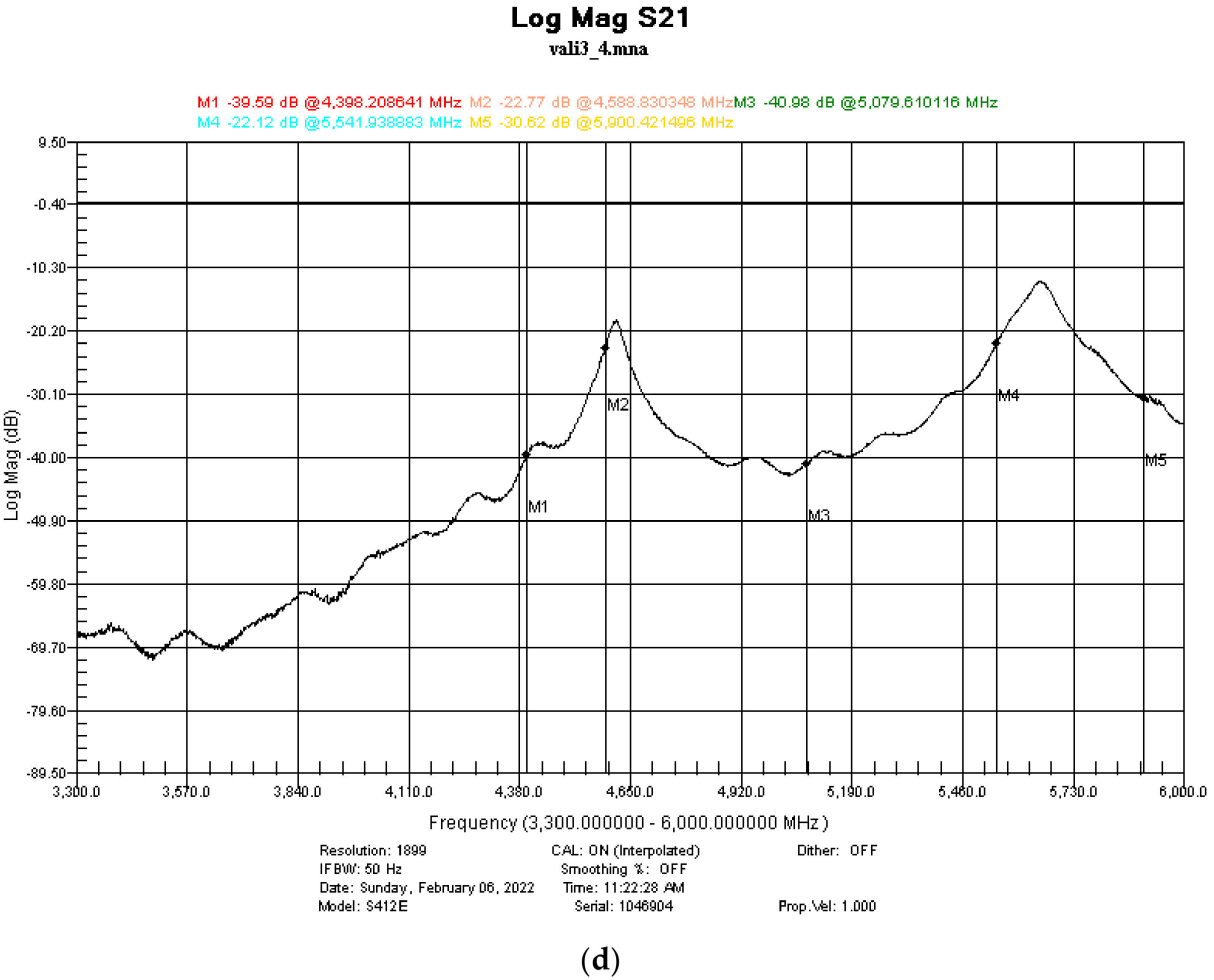
EMI shielding measurements in V sample and reference. (**a**) EMI-shielding measurement of reference; (**b**) EMI-shielding measurements on 1 layer of V sample; (**c**) EMI-shielding measurements on 2 layers of V sample; (**d**) EMI-shielding measurements on 3 layers of V sample.

The measurements were conducted on samples painted with 1, 2, and 3 layers of the respective paint applied, as shown in Figure 1 in the Experimental section. The first EMI-shielding measurements were conducted on the V samples. Examples of spectra are provided in Figure 8b–d.

**Figure 9:**
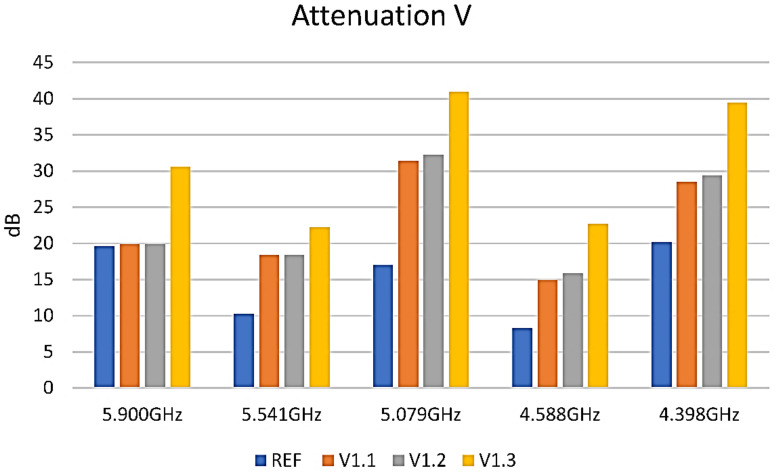
EMI-shielding measurements on V sample. Blue reference, orange 1 layer, grey 2 layers, yellow 3 layers.

The results can be more clearly seen in Figure 9. As can be observed, the third layer of paint shows a significant absorbance within the entire band measured. This is also in correlation to the measured resistance of the layers.

**Figure 10:**
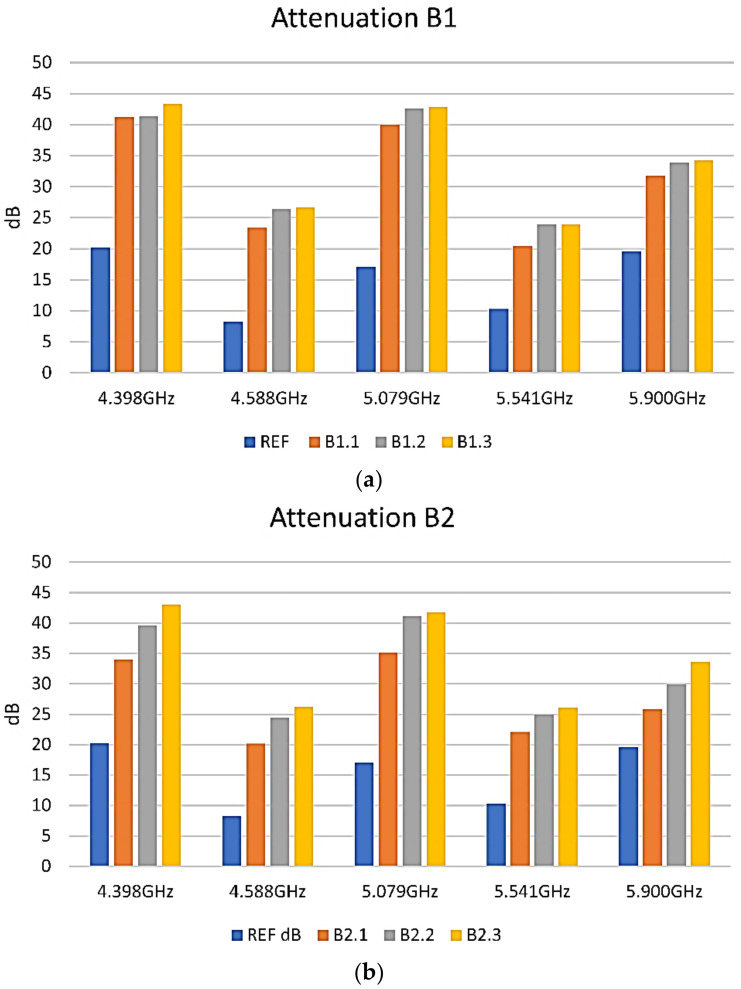
EMI-shielding measurements on B formulations samples. Blue reference, orange 1 layer, grey 2 layers, yellow 3 layers. (**a**) Graphic representation of EMI-shielding measurements results on B1 sample; (**b**) Graphic representation of EMI-shielding measurements results on B2 sample.

Then, samples were prepared using the B paints. Overall, attenuations from −25 up to −50 dB at some frequencies were also observed for the B formulations. Examples of shielding performance of the B1 and B2 paints are presented Figure 10. Regarding the B3 paint, this was proven to have a similar response to that of the B2 paint, and its behaviour was not included in the figure.

For the B1 formulation, it was observed that the difference in shielding between a different number of layers of paint is not significant, as observed in Figure 10a. However, for the B2 and B3 formulations, where similar behavior and shielding values were obtained, the number of applied layers was found to significantly affect the shielding performance, as shown in Figure 10b. Therefore, the material presents good absorbance properties.

**Figure 11:**
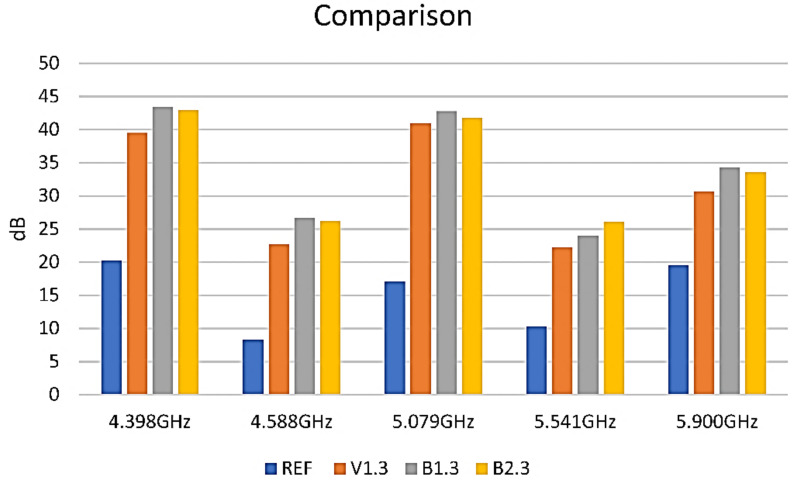
EMI-shielding measurements on 3 layers of each paint studied.

For the 3 layers of each formulation, a comparison of their attenuation at the specific marker frequencies is presented in Figure 11.

It could be observed that the V1-3, B1-3, and B2-3 samples are pretty similar in behavior and that the absorption of the composite layers seems to correlate strongly with their electrical resistance. The lower the resistance, the better is the absorption. 

Based on the structural characterization, we can assume that the presence of a high number of interfaces in the multicomponent composites promotes the interfacial polarization, which occurs on the carbon allotropes particles with relatively high conductivity. This leads to the accumulation of charges at interfaces and the generation of dipoles on semiconductive magnetite particles. The interfacial polarization and associated space-charge relaxation processes contribute to the EMI-shielding performance of the composites. To elucidate the shielding mechanism, further detailed study is ongoing. The most important issues of this study were finding the composition and the preparation method to obtain a high-shielding performance while at the same time maintaining ease of application and the development of uniform and stable surfaces. As a next step, the mechanisms will be studied in more details. 

### 3.6. Environmental Testing for Potential Commercial Applications

**Figure 12:**
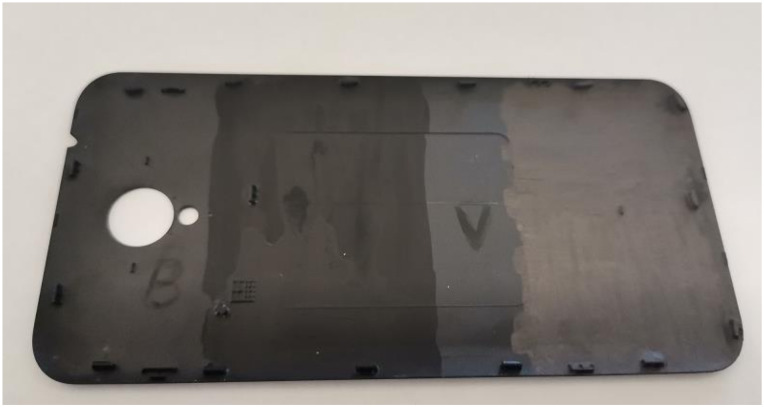
Cellular phone back side chassis coated with 2 paints samples.

The tests for commercial applications were conducted for two of the developed paints presenting the best shielding properties. The test was focused on potential applications to the cellular-phone industry, and the paint was applied, as illustrated in Figure 12, on the backside of a cellular-phone chassis.

The smart cellular phones are complex electronic devices that contain both analogue and digital circuits. People are carrying these devices wherever they go, many times accessing industrial sites with a high density of electromagnetic fields or nearby processes that are generating a broad spectrum of electromagnetic perturbations such as welding, plasma cutting, high power frequency inverters, etc. In addition, these devices could also be generators of electromagnetic fields and could disturb sensitive electronic equipment in laboratories. Thus, these devices are prone to EMI, and they were considered a potentially good application for testing the functionality of our paints onto a polymeric surface.

**Figure 13:**
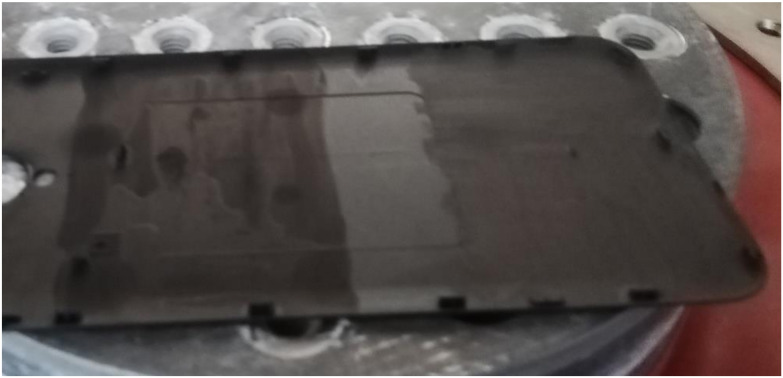
The sample coatings after two combined cycles of temperatures from +60 °C to −20 °C and relative humidity from RH 90% to 10% with 8 h maintaining time at higher and lower temperatures.

It was observed that both paint samples presented excellent adhesion properties, and despite the fact that the paint coating was applied with a paintbrush, the obtained layer was uniform, and no agglomerations occurred. In order to verify the behaviour of the paint under the stress of environmental factors such as temperature and humidity, two cycles of combined humidity and temperature challenges were conducted. In each cycle, the temperature was lowered to −20 °C and humidity (RH) at 10% and maintained there for 8 h, and then the temperature was raised to 60 °C and humidity (Rh) 90% and maintained there for 8 h (the method was presented in experimental section). As can be observed in Figure 13, after these cycles, the paints were not deteriorated, and no exfoliations occurred. 

**Figure 14:**
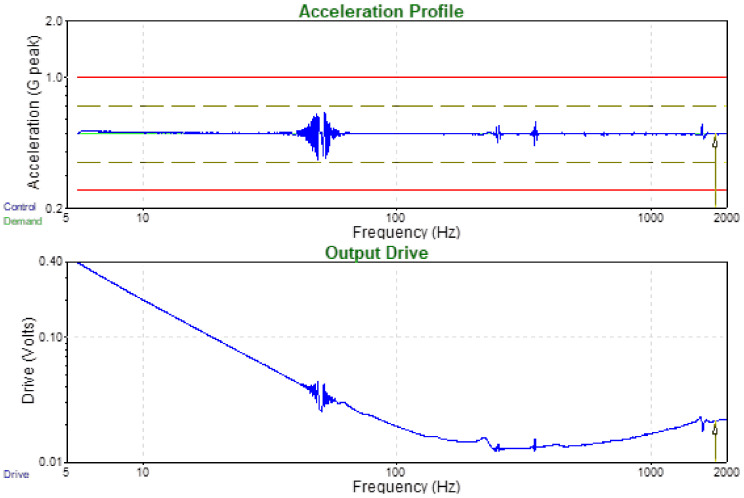
Acceleration profile and Output drive for the applied sine vibrations.

**Figure 15:**
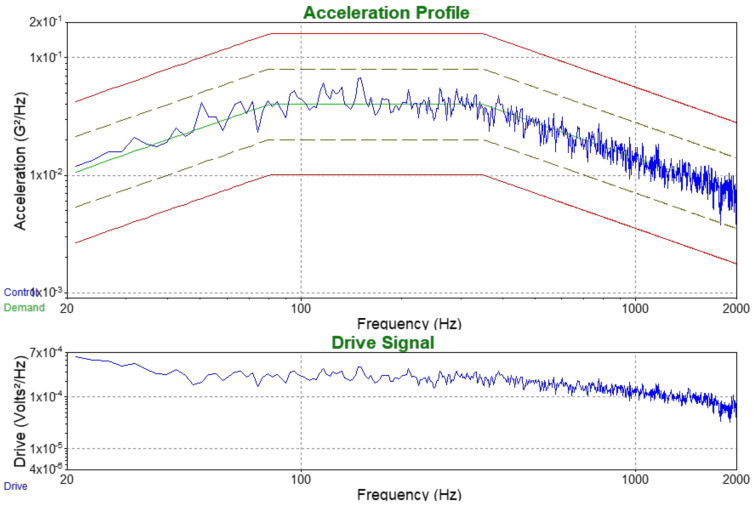
Acceleration profile and Output drive for the applied random vibrations.

The second step of testing of paints applied to the cellular-phone chassis was vibrations. Sine vibrations according to the specifications in Table 2 and Figure 14, and random vibrations according to 20 Hz to 80 Hz +3 dB/octave, rise to 0.04 g^2^/Hz; 80 Hz to 350 Hz at 0.04 g^2^/Hz; 350 Hz to 2 kHz −3 dB/octave, roll off and profile as shown in Figure 15 were used.

**Figure 16:**
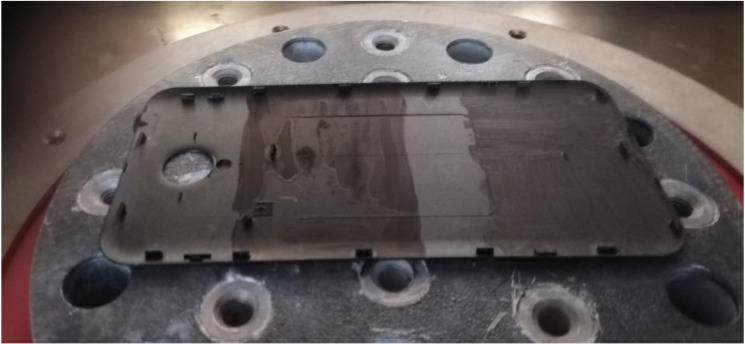
The sample coatings after vibrations.

In Figure 16, one can observe that both the V and B3 paint formulations had an excellent behaviour, and applied vibrations did not generate any cracks or exfoliations and did not affect the coatings.

## 4. Conclusions

Alcohol-based paint-like fluid mixtures based on graphite, carbon black, graphene, Fe_3_O_4_, Fe ore, and PEDOT:PSS in various combinations were prepared and studied as potential materials for electromagnetic-interference shielding (EMI) in electronic equipment. The trials were focused on the development of homogeneous paint-like fluids that could be easily applied to plastics and metallic surfaces with a paintbrush, leading to homogeneous, uniform, opaque layers, drying fast in the air at room temperature; these layers had a reasonably good electrical conductivity and, subsequently, an efficient EMI-shielding performance. The structural and morphological characteristics of the coatings based on these paints indicated that these are not simple physical mixtures but hybrid composite coatings with particular properties. Increasing the number of applied layers resulted in more compact, homogeneous composite coatings with improved conductivity, due to better assembled carbon-based building-blocks components at a microscopic level. The EMI-shielding performance of these paints’ formulations of over 50 dBs proved to be comparable or even better than that of already existing market solutions that show EMI attenuations from 5 to ~37 dB with a few exceptions going over 50 dB. The environmental and mechano-climatic tests conducted on coatings onto cellular-phone chassis demonstrated that these paints pass the technology-readiness level (TRL) 5—technology validated in a relevant-environment (industrially relevant environment in the case of key enabling technologies) stage, a fact that places them only two steps away from the competitive manufacturing level. In conclusion, it was demonstrated that the developed paints could be effectively employed in commercial applications and be used in quite challenging environmental conditions.

## Figures and Tables

**Table 1:** Resistance measurements results.

Paint	Resistance (ohm) ± 0.01
B1.1	322.10
B1.2	142.90
B1.3	60.12
B2.1	217.30
B2.2	156.80
B2.3	66.09
B3.1	215.10
B3.2	158.60
B3.3	69.01
V1.1	336.37
V1.2	148.70
V1.3	70.80

**Table 2:** Sine vibrations specifications.

Frequency	Amplitude	Durate
5–2000 Hz	0.5 (g)	2 (Oct/min) (up)

## Data Availability

The raw and processed data required to reproduce these findings cannot be shared at this time due to technical or time limitations. The raw and processed data will be provided upon reasonable request to anyone interested anytime until the technical problems are resolved.
